# ‘I’m tired of justifying why my life has stopped’: A qualitative analysis of the experiences and support needs of young people caring at the end of life and into bereavement

**DOI:** 10.1177/26323524261442209

**Published:** 2026-05-12

**Authors:** Emily Harrop, Zoe Abbott, Noreen Hopewell-Kelly, Silvia Goss, Charlie Byrne, Jane Bayliss, Kerry Jones, Oliver Clabburn, Rachel Warren, Alison Penny, Helen Leadbitter

**Affiliations:** 1 Marie Curie Research Centre, School of Medicine, Division of Population Medicine, Cardiff University, UK; 2 School of Health, Wellbeing & Social Care, Open University, Milton Keynes, UK; 3 Oliver Clabburn Social Research, Taunton, UK; 4 Marie Curie, London, UK; 5 Childhood Bereavement Network/National Bereavement Alliance, National Children’s Bureau, London, UK; 6 Young Carers Initiative, London, UK

**Keywords:** young carers, family caregiving, grief, bereavement, palliative care, qualitative, children and young people

## Abstract

**Background:**

Family carers play an essential role in caring for terminally ill relatives, but evidence regarding the experiences of young/young adult carers is limited.

**Objectives:**

To explore the experiences and support needs of young/young adult carers when caring at the end of life and into bereavement, and identify ways of improving support. In the ‘Discussion’, we apply the Dual Process Model of coping with bereavement (DPM) to help conceptualise our findings.

**Design:**

A mixed-methods study comprising; an online survey of statutory, voluntary and community sector professionals/volunteers working with current or bereaved young/young adult carers (*n* = 106); two focus groups with professional survey participants (*n* = 8); three focus groups with bereaved young/young adult carers (*n* = 9). Here, we describe our qualitative themes following reflexive thematic analysis of free-text survey and focus group data.

**Results:**

Our three main themes centre around; providing care at the end of life; navigating bereavement; and managing daily life and relationships. When caring, young/young adult carers struggle with a lack of emotional and practical preparedness for death, caregiver burden and changing roles and relationships within the family. Post-bereavement, they experience complex emotions relating to the death and the loss of their caring role/identity, alongside difficulties dealing with administrative/practical tasks and changes to living situations and family roles. Pre/post-bereavement young carers experience difficulties opening up and seeking help and lack of understanding or support from family, peers and schools/colleges, whilst also struggling with school attendance and performance. Transitioning between education and work/unemployment creates support gaps and needs unique to young adult carers, alongside a general lack of preparation for adult life.

**Conclusion:**

Young/young adult carers experience multiple challenges and support needs, which can be usefully theorised using an adapted version of the DPM. Cross-sector changes, reflecting public health approaches, are needed to improve the informal and formal support available to young people when caring and bereaved.

## Introduction

Family carers play an essential role in caring for terminally ill relatives. However, most evidence relates to adult experiences,^1–5^ with much less known about the experiences of young carers and young adult carers. Young carers are defined as children and young people under the age of 18 who provide ongoing care and support to a family member who is physically or mentally ill, disabled or misuses substances.^6,7^ Young adult carers represent a slightly older age group, commonly defined as aged between 18 and 25.^6,7^ Studies in several countries, including the United Kingdom, report that between 2% and 8% of young people may be a young carer,^7,8^ with females and low-income families disproportionately affected.^8,9^

Young carers have specific legal status and recognition in England^10,11^ and other UK nations.^12–15^ However, this group of carers has been described as remaining ‘hidden’ and unrecognised in their roles, reflecting a gap between the provision of the law and its implementation,^7,8,16^ and the under-identification of ‘young carers’ within families.^17^ Although some studies have identified positive psychosocial outcomes for young carers, such as stronger family relationships and empathy,^7,16,18–20^ negative consequences include higher prevalence of mental health problems, social isolation and emotional and educational difficulties.^8,17^ Research has also shown that childhood bereavement negatively impacts educational experiences and outcomes,^21–24^ and mental health with estimations that 5%–20% of bereaved children and adolescents develop psychiatric difficulties.^25,26^

In the wider literature on young carers, impacts of caring have been shown to include additional responsibilities and tasks (such as household tasks, physical, personal and emotional care and taking care of siblings), impacts on schooling and social activities, as well as financial constraints.^18,27,28^ It is also recognised how becoming a young carer often coincides with already challenging life-transitions – from child to adolescent to young adult,^17^ with studies commonly describing loss of childhood due to growing up too quickly.^17,29^ The support that young carers and bereaved young people have around them from families, peers, communities and schools is vital for helping them to cope.^17,29–31^ However, lack of open communication within families,^29,32^ challenges maintaining parenting roles after the death,^33^ and the difficulties of bereaved children and young people expressing their feelings^33^ have been documented. Support from schools has also been shown to be limited by lack of awareness of the effects of being a young carer, of being bereaved and of how to identify and support such students,^30,34^ alongside poor communication and information sharing, inflexible systems and variable access to specialist emotional support.^30^

Research focused specifically on children and young people caring for family members with terminal illness is more limited,^8,9,17^ in particular for non-cancer diagnoses.^18,20^ Within this relatively small body of research, various challenges relating to communication with professionals, navigating healthcare systems, being involved in end-of-life decisions and anticipatory grief have been described.^8,16–18,20^ Young carers have been shown to receive less or inadequate information and education on the condition and care of their relative, and less support and acknowledgement than adult caregivers, leaving them feeling marginalised and excluded,^8,16–18,29^ and reporting worse experiences of direct care and a perceived need for more assistance with their grief.^35^ Within families navigating terminal illness, research has demonstrated tendencies to protect children and young people by not talking about terminal prognoses or the death,^29,36–39^ uncertainty about how to prepare children for the death^20,30,33^ and breached moral expectations caused by needing to prioritise the care of a sick parent over their children.^40^ A lack of needed support from peers at these difficult times has also been described.^29^

Although initially developed in relation to adult spousal grief following bereavement, the Dual Process Model of coping with bereavement (DPM)^41^ can be applied to all grieving or loss experiences.^42^ The model posits that bereaved people oscillate between two orientations and types of coping. Loss-oriented coping focuses on processing the difficult aspects of the loss, in particular loss of relationship, and includes grief work, ruminating and yearning for the deceased person. Restoration-oriented coping relates to the practical and psychosocial changes that occur as a result of the bereavement, coping in everyday life and building new roles and relationships.^41,42^ Although less applied to bereavement in childhood,^43^ and pre-bereavement experiences,^44^ the apparent relevance of the model for conceptualising the practical, psychosocial and emotional challenges experienced by caregivers has been suggested,^44^ including specific application to the experiences of bereaved adolescent caregivers of parents with HIV in Nigeria.^45^

Using qualitative survey and focus group data collected from bereaved young/young adult carers, and the professionals/organisations who support them, our research aimed to explore the experiences and needs of young people when caring at the end of life and into bereavement and identify ways of improving the support available to them, adding to the limited evidence on this under-researched population. By retrospectively applying the DPM^41,42^ to our findings in the ‘Discussion’, we also adapt and extend the application of the model, suggesting a useful framework for conceptualising the pre- and post-bereavement experiences of these groups of young people.

## Methodology

We conducted a mixed-methods study comprising:

- An online cross-sectional open survey of statutory, voluntary and community sector professionals or volunteers working with current or bereaved young/young adult carers.
- Qualitative focus groups with bereaved young adult carers and focus groups with professionals who work with current and/or bereaved young/young adult carers.

In this paper, we describe our qualitative analysis of free-text survey and focus group data, informed by our phenomenological interest in exploring how people make sense of and give meaning to their lives and experiences. Reflecting this interest, we followed the principles and practices of reflexive thematic analysis^46^ and the corresponding Reflexive Thematic Analysis Reporting Guidelines (RTARG)^47^ (Supplemental File 1).

*Survey with professionals*: The survey was administered via the online survey platform JISC (https://www.onlinesurveys.ac.uk/) and was open between 8 October 2024 and 5 March 2025. It was disseminated to a convenience sample through the communication channels of relevant organisations and networks (e.g. illness, bereavement, young carer and children’s organisations, education, health and social care networks), using e-mail bulletins, newsletters, distribution lists and organisational and personal social media platforms (e.g. X, Facebook). The survey included study information at the start of the survey and click through consent indicating agreement with the study information and consent to participate in the research (see Table 1, study inclusion criteria).

**Table 1.** Study inclusion criteria.

Data collection activity	Inclusion criteria	Exclusion criteria
Survey and professional focus groups	• Health, social care or other professional/volunteer • Experience of supporting young carers and their families at the end of life and/or in bereavement, or a representative of specialist young carer service or organisation • Aged 16 or above • Based in the United Kingdom	• Service-based outside of United Kingdom
Bereaved young carer focus groups	• Self-identified as having a previous role caring for a family member/relative in the United Kingdom • Bereaved of the cared-for person within the last 3 years, when aged 25 or under • Aged 16 or over	• Aged under 16 • Aged over 25 at time of bereavement • Unable to provide informed consent

The (non-validated) survey was designed by the research team, which included palliative care and bereavement researchers, representatives from palliative care, bereavement and young carer organisations and two public representatives with personal experience of caring for a family member at the end of life and bereavement. Questions were informed by study aims and previous research by team members involving a survey of bereavement services.^48^ Open and closed questions covered descriptive information about the services/organisations, support provision, perceived challenges experienced by young carers, perceived challenges for services/organisations when supporting young carers, examples of good practice and suggestions for how best to improve support for young carers (Supplemental File 2, Survey Template).

*Focus groups with professionals*: To explore survey topics in more depth we conducted two follow-up focus groups with a sub-group of professional survey participants, inviting all who agreed to be contacted about taking part in a group (*n* = 60) with a choice of two dates in February and March 2025. All participants who responded positively to the invitation were offered a place in the focus group of their choice.

*Focus groups with bereaved young carers*: We conducted three focus groups with bereaved young/young adult carers, exploring experiences, perceived support needs and ideas for change, when caring and following bereavement (see Table 1). These participants were recruited via adverts disseminated through the organisations and channels described above, as well as staff and student networks at universities connected to the research team. Participants in the young carer groups were offered £20.00 Love2shop vouchers to reimburse them for their time. Dates for the groups were arranged at mutually convenient times when at least three people had ‘signed-up’ for a group. The three groups were held in January, June and July 2025.

Interested participants in the young carer and professional groups were sent a participant information sheet, and all participants gave written informed consent before the focus groups took place. We conducted the discussions on MS Teams using semi-structured topic guides developed by the team, including our public representatives (Supplemental File 3, Topic Guides). All focus groups lasted an hour, and were facilitated by experienced qualitative researchers N.H.K. and E.H., and H.L. who has extensive experience of working with young carers. Fieldnotes were taken by E.H. to inform analysis, with short ‘de-brief’ sessions immediately following the focus groups enabling facilitators to give initial reflections on these discussions. Focus groups were auto-transcribed on MS Teams, checked for accuracy and anonymised prior to analysis by N.H.K. The identifiers assigned to these participants reflect both the source of the data collected and unique participant number, for example, YCFG1 YC3 (young carer focus group 1, young carer participant 3), PFG1 P4 (professional focus group 1, prof. participant 4).

Qualitative data were analysed using NVivo 14 following the principles and practices of reflexive thematic analysis.^46^ Three separate NVivo files were created for the coding of qualitative survey data, young people focus group and professional focus group transcripts. After initial reading, annotation and discussion of free-text data and transcripts by Z.A. and E.H., Z.A. coded all data in NVivo. Z.A. began by generating inductive codes which closely described individual participant experiences or perspectives, before creating broader codes which meaningfully described shared or similar experiences, and organising and connecting these under higher-level analytical categories. This was an iterative process in which Z.A. moved back and forth between the data and analytical concepts, discussing interim and final versions of the coding framework with E.H. and preliminary themes with the wider project team. Throughout this process, the different professional and experiential knowledge and backgrounds of the team (including our public representatives) informed our discussions and interpretations of the meaning and significance of the data, primarily through presentations and discussions at team meetings. An overarching framework and thematic narrative was created by E.H. and reviewed by all authors. This framework brought together key themes and sub-themes from the separate datasets under common analytical headings, while retaining separate descriptions and discussions of descriptive themes by participant (professional vs young carer) backgrounds.

## Participants

### Survey participants

A total of 106 surveys were completed. Most participants worked either with a dedicated young carers charity/NGO (42.5%; *n* = 45), a palliative care service (14.2%; *n* = 15) or a bereavement service or charity (12.3%; *n* = 13). A smaller number of participants worked with either a children’s (7.5%; *n* = 8) or an illness-specific charity/NGO (6.6%; *n* = 7), a local authority (7.5%; *n* = 8) or in the education sector (6.6%; *n* = 7; other: 2.8%; *n* = 3). Participants held a broad range of job roles, including most commonly manager/director (23.6%; *n* = 25), team manager/coordinator (23.6%; *n* = 25) and support worker (19.8%; *n* = 21). The 106 participants came from 90 different services/organisations. Most (83.3%; *n* = 75/90) provided regional support in specific geographic areas across the United Kingdom, most commonly within South East England (*n* = 19) and Wales (*n* = 12). A smaller number offered support either UK-wide (*n* = 11) or nationally across all of England (*n* = 4), Scotland (*n* = 1), Wales (*n* = 3) and Northern Ireland (*n* = 1). All participants provided free-text responses to survey questions included in this analysis.

### Focus group participants

The two professional focus groups (each *n* = 4) included two managers, four practitioners, one school’s engagement worker and a network director at a sixth form college. They were employed at adult hospices (*n* = 2), young carer organisations (*n* = 2), a children’s hospice (*n* = 1) and a sixth form college (*n* = 1), and were located in England (*n* = 5), Wales (*n* = 2) and Scotland (*n* = 1).

The three young carer focus groups (*n* = 5; *n* = 2; *n* = 2) included five male and four female participants who ranged in age from 18 to 26 (median 20). Between them they had cared for, and been bereaved of mothers (*n* = 4), fathers (*n* = 2), siblings (*n* = 2) and grandparents (*n* = 2), whose illnesses/conditions included cancer (*n* = 5), dementia (*n* = 2), life-limiting/developmental (*n* = 2) and chronic conditions (*n* = 1). One participant had lost both parents within the same year. Participants were between approximately 6 months and 3 years bereaved at the time of recruitment (median approximately 1 year; missing data, *n* = 2).

## Thematic narrative

We created three main themes to describe the challenges and support needs of current and bereaved young/young adult carers. These centre around; providing care at the end of life; navigating bereavement; and managing daily life and relationships (Table 2).

**Table 2.** Themes and sub-themes.

Themes	Sub-themes
Providing care at the end of life	• Inadequate communication/support from healthcare staff • Caregiver burden and responsibilities • Changing family roles and relationships • Lack of emotional and practical preparedness • Improving support for young carers at end of life
Navigating bereavement	• Loss of relationship and caring role/identity • Complex emotions and grief • Difficulties dealing with administrative and practical tasks • Changes to living situation and family roles • Improving practical and emotional support with bereavement
Managing daily life and relationships	• Difficulties with relationships/support from family • Difficulties with relationships/support from peers • Impact on educational experiences • Lack of support/sensitivity from schools/colleges • Transitioning into adult life • Improving school-based support • Supporting transitions

### Providing care at the end of life

This theme captures the multitude of difficulties experienced by young people when caring for terminally ill family members, including inadequate communication/support from healthcare staff, high levels of caregiver burden and responsibilities, changing roles within the family and lack of emotional and practical preparedness for death. Participant views on the changes needed to address these difficulties are also described.

In terms of healthcare support, and related to this caregiver burden and responsibilities, professional participants explained how family reluctance to, or difficulties accessing the right levels of healthcare support could leave young carers with high levels of responsibility for their family members care and medication. At the same time, they could also be excluded from care discussions with healthcare teams, leaving them under informed about their relative’s changing condition, causing stress, anxiety and high-risk of burn out. These issues are compounded in more complex caring situations, such as parents with addictions, while the added caring responsibilities that the young person may need to take on for other family members such as siblings was also noted.

We had a teenager, teenage girl changing her 10, 11-year-old brothers nappies and stuff like that. And you know if you talk through that with families then they understand and usually in by far the majority of cases that’s very easily resolved. (PFG 1, practitioner, young carers service, P4)

Our young carer participants described being closely involved in the care of their family member, which included parents, grandparents and siblings. They also described their experiences of changing roles and relationships within their families as the illness progressed, and the strain that this sometimes caused. Financial pressures meant that parents often needed to keep working, with the young person taking on greater responsibility for younger siblings and managing the care needs of the unwell family member. They experienced a sense of growing up too fast and missing out on their childhood as a result of these additional responsibilities, describing the emotional impact of feeling ‘the parent being stolen from you’ (YC6), by the treatment as well as the illness.

You have to be strong because one parent is sick and the other is busy working to cater for your needs. So, you have like when Mum is going for treatment you have to explain to the siblings. She’ll be alright. You have to comfort them. When though yourself, you’re really struggling to understand the situation, will [it] be all right? (YCFG1, daughter YC3)

The lack of emotional and practical preparedness for death and bereavement that was described similarly relates to both healthcare support and family relationships. Professional participants described the masking of terminal prognoses by family members and explained how difficulties experienced by parents and other professionals in having honest conversations about the illness made it harder to emotionally prepare children and young people. Hospice-based participants also explained how the presence/needs of children and young people were not routinely assessed in initial palliative care assessments, and were often invisible to community nursing teams who visit during the day, limiting the support that they could offer them.

I feel the young person feels very lonely and overwhelmed at this prospect. Challenges I have encountered especially around parents who are dying of cancer is they do not want to give up hope, so they do not always want us to do pre bereavement work with the child as they don’t want it to be real I suppose. (Support Worker, Young Carers Organisation, survey ID8)

A young carer participant also described unhelpful attitudes and behaviours of healthcare professionals as his mother’s death approached. He explained that having cared for her from a young age, he was well informed about her condition and needs, and was deeply frustrated at not being fully involved in discussions about her condition and care (YC6). Other young carer participants described not knowing what to expect around the time of death, and finding the uncertainty and unpredictability associated with this phase of illness difficult to manage, causing them significant anxiety, including in one case severe separation anxiety (YC8).

Having been a young carer since I was about 5, all the way up until just a couple of years ago . . . I knew my mum’s health conditions better than she did. I knew them better than 90% of the medical professionals that saw her . . . So when there were people trying to talk to me and they were sort of daintily talking around the medical topics or not quite believing that what I was saying was accurate or that I truly understood what I was saying, it was even more infuriating . . . . (YCFG2, son YC6)

To address these difficulties, professionals stressed the importance of raising awareness of the circumstances and needs of young carers in healthcare contexts at the end of life, and becoming better at formally identifying young people in these situations, including through the standard assessment practices of palliative care professionals. Some described the benefits of using collaborative communication techniques with young carers and the importance of working in a family/person-centred way to assess need and build communication skills within families. This need for more honest communication between healthcare professionals and young carers was similarly discussed in the young carers group. Young carer and professional participants also emphasised the need for access to counselling services, and better information and guidance on what to expect and the more practical aspects of preparing for a death, including legal advice.

Maybe some information that we can find online on what to expect when a loved one dies and you know . . . planning a funeral. And you know what to expect to happen when you’re planning it so that you can kind of mentally prepare yourself because for me it was like they were showing me a shoe catalogue. But it was coffins instead and it was very strange. (YCFG3, sister, YC8)

### Navigating bereavement

This theme describes the many difficulties experienced by young carers following the death of their cared-for relative, in particular the loss of their caring role/identity, the complex emotions often felt and the challenges of navigating administrative/practical tasks, changes to living situations and family roles. Suggestions for improving practical and emotional support for bereaved young carers are also captured.

The disruptive impact of bereavement on young carer identities was widely described by professional participants, who explained how their heightened sense of loss was caused not only by the loss of relationship but also the loss of (sometimes long-term) caring roles. Other emotional complexities noted by professionals included the traumatic effects of witnessing suffering and, in some cases, death, as well as feelings of guilt and confusion. Professional focus group participants elaborated on the ‘surprise’ emotions experienced by bereaved young carers – such as disappointed expectations for more parental attention following the death of siblings, feelings of relief from no longer witnessing suffering or needing to care, coupled with the guilt of feeling this way.

The adjustment period following a bereavement can be a challenge, not only are they experiencing a loss, but also some young carers have not known anything differently other than helping to care for their family member and so they may feel a bigger sense of loss. (Manager, Young Carer Org, survey ID17)

A number of young carer participants also described their sense of loss relating to their caring role and identity, and the changes that this brought to their daily lives and routines, alongside grieving and coming to terms with the death of their family member. Other emotional difficulties included feeling ‘scared’ (YC2) over their future prospects, and persisting separation anxiety. Feelings of guilt were multi-dimensional, and related to whether more could have been done to help their relative, their relief that their relative was no longer suffering, that their responsibilities had ended, and feeling happy again.

. . . their care needs are an essential part of our reality. So suddenly we almost lose a huge part of ourselves . . . You look for that caring thing that you did before . . . And there is so much guilt that can exist . . . Why didn’t I see it? Could I have done something you know? Could I have made it easier? All of that? And then there’s the guilt of starting to feel a little bit better about the loss. But there’s also the guilt that comes from, even if it is only very brief, sudden relief that you’re no longer responsible. (YCFG2, son YC6)

A number of more practical challenges were also described by professional and young carer participants, in particular relating to the administrative tasks that need to be managed following a death. Examples given by our young carer participants included the pressure of making funeral arrangements and getting it ‘perfect’ (YC1), managing financial changes relating to pensions and benefits, informing personal contacts and organisations of the death, frustration with the ‘Tell Us Once’ death-notification service for government departments, and receiving continued correspondence for their deceased relative. They described receiving minimal guidance for these tasks, needing to figure them out for themselves, and found them burdensome and an unwelcome distraction from dealing with their emotions at this very difficult time.

But like there’s so much like stuff you have to think about . . . even now I still actually don’t know what I have and haven’t done . . . And so we were sort of like blindly fumbling at trying to work out like how do you close someone’s bank account and stuff like that? . . . Was the stuff that I just didn’t have time for either as well because I wanted to process my emotions and also the last thing you want to be doing is chasing a bank and proving that your dad’s died over and over again . . . . (YCFG1, daughter YC4)

Professional participants commented on the further complications and difficulties that could be caused to bereaved young carers by loss of household income and changes to living situation, including in some cases going into care or needing to take on responsibility for younger siblings. Two of our young adult carer participants who were left living alone in their family homes after the deaths of their parent and grandparent provided insights into the impacts of these changes, describing how hard they found this adjustment – practically and emotionally, with one needing to temporarily move in with another relative.

I felt like I just needed to run away from that reality, so I left. I had to go stay with my sister a bit because I was the only one staying with my grandfather for quite a long time. I never really had the house to myself like that. So when he eventually died, I couldn’t just be the one staying at. . . I really couldn’t take it for some time. (YCFG3, grandson, YC9)

Reflecting on these difficulties professional respondents and young people discussed the importance of improving the emotional and practical support and information available for recently bereaved young carers. Several young people described benefiting from counselling support, which was seen to be particularly helpful for managing complex emotions, whilst specialist support with dealing with separation anxiety was also recommended. One young carer participant described a need for more guidance about common grief reactions and emotions, with some also appreciating or wanting opportunities for peer support with other bereaved young people who could relate to their experiences.

I’d love to have been told that you’re going to feel every single emotion under the sun and every single emotion is OK. And it’s to be expected that you’re going to . . . be happy again and you’re going to feel guilty about it, but that’s OK. . . . Yeah, I think just acceptance that so many feelings are gonna happen. That’s normal, and it’s part of grief. (YCFG2, sister, YC7)

Young people also discussed needing more guidance on bereavement specific tasks such as obtaining death certificates, contacting different agencies and handling coroner’s processes, as well as on more general life skills such as managing money, benefits, finding work, progressing in higher/further education and in one case managing veterinary care for family pets.

Maybe some sort of education or training or not training, but something like that in terms of a class on adult life or just covering some sort of things for a young person to maybe navigate themselves. I was very fortunate to have my two older sisters who are fully functioning adults and know, know quite a bit but . . . . (YCFG1, son YC1)

### Managing daily life and relationships

The practical and emotional challenges of caring and bereavement were widely recognised to impact on other areas of life. This theme describes the challenges navigated by young carers pre- and post-bereavement with regards to their relationships with family and peers and in their everyday education and/or (un)employment settings, alongside related suggestions for change.

With regards to relationships with family and friends, professional participants commented on the tendencies of children to try and protect their family members by hiding their feelings, as well as the already noted tendencies of adult family members to conceal information from children. These difficult family dynamics, alongside a lack of peers with shared experiences to talk with, were felt to leave young carers feeling isolated and overwhelmed and lacking the emotional support that they needed from their family and friends.

Protecting their family from their fears and feelings leads to feelings of isolation, loneliness. Not being able to speak with peers who are going through something similar adds to this. (Team leader, Illness charity, survey ID58)

Family support and relationships were also discussed by young carers, who although described the importance and benefits of mutual support within families, also gave examples of ‘support’ that was less helpful. Some described hiding their feelings to avoid upsetting other family members and reflected on how different grieving responses within families can hinder family-based support. YC8 recalled feeling pressured by her grandparents to behave better at school to avoid burdening her parents during her brother’s illness, and reflected on how such attitudes could lead to resentment and tensions within families. Some perceived a lack of understanding or genuine empathy, and in one case unwanted pressure to take up formal services such as counselling or other ‘healing’ activities, which threatened this bereaved son’s sense of control over his grieving.

When you’ve lost your mum . . . you’ve lost control over a lot of things in your life. The one thing that I had control over was how I grieved, but despite that, a lot of people in these caring positions were trying to almost take my grief away . . . It wasn’t like I was trying to be fixed and you know, I don’t think you really can be fixed from grief . . . I want to grieve and I want to feel these things and I'll decide how I go about them. (YCFG1, son, YC5)

A perceived lack of understanding amongst peers also limited the extent to which these young people felt supported, with participants describing how friends avoided talking about their situations due to not knowing what to say or assumptions that they were ok and would rather not talk about it. Some participants explained how they found the cliched and constant use of the word ‘sorry’ unhelpful, ungenuine and triggering of their pain and grief, while YC2 described the ‘turning point’ enabled by his friend taking a genuine interest which helped him to share his feelings.

Societal sort of assumption that like oh well, you know, (YC2) is . . . just sort of soldiering on and, like sort of keeping going. He’s probably fine, he probably just doesn’t want to be spoken to, but . . . when that one person came to speak to me and like, I felt like they were genuinely interested in what I had to say . . . I think that was like sort of a turning point for me, and it then made me feel like I could actually sort of voice what I was feeling. . . . (YCFG1, grandson YC2)

Professionals and young carers made a number of suggestions and recommendations for improving the support available from friends and family. As already noted, some professionals described the importance of building communication skills within families, while the family focused bereavement support received by YC7 was seen as helping to improve the mutuality of support and understanding within her family. Professional respondents valued opportunities for young carers to build friendships with other young carers, and to be given breaks from caregiving to enable participation in social activities. Including grief education on the school curriculum was also viewed by young people and professionals as an important way of developing more understanding and supportive communities, helping both the bereaved and those around them to engage in supportive conversations.

I’ve had so many sort of retrospective conversations with my friends where they’ve said I don’t know what I would have said, so I haven’t said anything . . . And I think we need to be teaching people, especially young people, who are being bereaved when they’re sort of in an already difficult stage of their life how to be having those conversations with their friends or relatives or whoever it might be? (YCFG1, daughter YC4)

With regards to educational experiences, the impacts of caring and bereavement on a young person’s ability to manage the demands and expectations of school, college and university were widely noted. Professional participants explained how attending and completing school or college work was difficult for young carers due to the competing care needs of their family members, and reluctance to leave them, while their grief and emotional responses to loss makes it hard to continue ‘as normal’ following the death. The impact of caring responsibilities on maintaining friendships and the confidence and self-esteem of young people were also noted, alongside problems with bullying and stigma associated with both caring and bereavement.

Trying to combine role of young carer alongside managing school, other siblings sometimes, financial stress/emotional stress, worry about losing the family home, what will happen to them after the person has died. (Palliative care social worker, hospice, survey ID68)

Several young carer participants described similar negative impacts on their educational experiences, especially towards the end of life and following the death. They described an increasing reluctance to leave their unwell sibling or parent as the illness progressed and extended periods of absence from school. School was experienced as very tiring when dealing with ‘turmoil inside your head’ (YC8), with perceived hostility from peers only making things worse. A fear of leaving her seriously unwell brother gave YC8 separation anxiety, leading to a formal diagnosis of post-traumatic stress disorder (PTSD). Negative impacts on school work were also described, as well as difficulties thinking about future plans, including the effects of missing out on guidance from an incapacitated or deceased parent.

When I had some time out of school, because I didn’t go to school for a bit, they got the school charity (name) involved and that was nice . . . it was just an hour where all the people that didn’t know how to be around me left me alone and we just did art in this room. And then I went back to the horrible corridors and tried not to scream at everyone. (YCFG3, sister, YC8)

These difficulties managing school/college-life were seen to be exacerbated by various limitations with the support provided within these settings. Professional survey and focus group participants considered the specialist support available within schools to be inconsistent and highly variable across schools, while a more general lack of understanding or flexibility towards current and bereaved young carers was noted. Examples were given of young carers being required to retell their stories multiple times, rigid attendance policies and fines being applied to families (e.g. for frequent or extended periods of student absence), and schools ending specialist emotional support for a young person post-bereavement. It was also explained how a child or young person’s desire for normality and to fit in, coupled with stigma and concerns over bullying can discourage families from informing or accepting support through school. Acceptance of caring as a normal part of family life and/or beliefs that they should prioritise the needs of their cared-for family member over their own, including ‘protecting’ them by not revealing their caring role, also mitigated against self-identification as ‘carers’ and associated help seeking.

Schools vary in the amount of support they offer for pupils as young carers/bereaved young people. As a young carers service, we are often advocating for young people in school and with teachers as they are often inflexible and not very understanding. School policies are a barrier to young carers needs being met, particularly attendance policies and attendance fines. (School Engagement Officer, Carer Organisation, survey ID40)

Some young carer participants also described a lack of understanding or flexibility within schools, including unrealistic expectations for how quickly they should ‘move on’ following the bereavement and lack of sensitivity when teaching death-related subjects on the curriculum. YC8 described finding the school environment ‘unsafe’ and gave an example of being unfairly punished in relation to unfulfilled homework tasks. She explained how it was only when her PTSD was diagnosed that her school responded to her needs, but that they were too quick to withdraw these extra accommodations when she appeared to be coping better. YC5 described a similar ‘uphill battle’ in the sixth form, and an unrealistic expectation from school that his academic work should be prioritised over his caring commitments.

I was already struggling before my mum got sick at the start of year 13. So obviously like school, one of the first to get told and for the entire time I kind of felt like I was fighting this uphill battle . . . none of the teachers really understood that school wasn’t exactly my priority at the time . . . They kind of had this, this approach that if I would just sort of like focus and do my work, that would make a lot of difficulties easier. (YCFG1, son YC5)

On leaving education around the time of his bereavement, lacking qualifications and opportunities, the same participant experienced further frustrations claiming job seekers allowance by having to repeat his story at every appointment, illustrating some of the specific challenges faced by young carers transitioning into adult life. Other similar examples were given by YC4 – whose father had died as she was finishing university, and who reflected on the invisibility of her grief due to her being outside of both education and employment, and YC1 – who felt that his caring responsibilities went unrecognised by those around him who expected him to continue with normal day-to-day life despite feeling ‘lost’ and that his life was ‘on hold’. Participants agreed that these transitions between education and work reflected support gaps unique to their age group, which also coincided with already described problems relating to lack of guidance or preparation for adult life and managing a household. A lessening of support for young/young adult carers when they leave school was similarly noted by some professional survey and focus group participants.

I got a job four months after my dad had died. And I think there was that assumption that like when I was going to get a job. So (YC4) is probably fine. And the fact that like that was still the only thing in my head . . . And so I think like a better understanding of bereavement in any form would benefit everyone who’s bereaved because once you understand more about it, I think you can handle it from a different perspective. (YCFG1, daughter, YC4)

To tackle these problems professional participants described a number of changes that were needed in schools, colleges and beyond. These included requirements for school bereavement policies and training, greater awareness and sensitivity of school/college staff to the circumstances and needs of current and bereaved young carers, including identifying them and enabling access to extra support, and greater allowances and flexibility in school policies regarding attendance and school-work/exams. There was also a suggestion for greater use of contextual offers across universities in acknowledgement of the impact of caregiving and bereavement on grade attainment. Some positively described initiatives which recognise and raise awareness of young carers in schools/colleges, such as topical assembles and award schemes, as well as young carer ‘champions’ and efforts at improving internal communication to help prevent the need for frequent retelling of stories. To address problems with stigma one hospice-based focus group participant who provided support in schools explained how they had moved to offering lunchtime or after-school appointments so that the young person was not so obviously receiving support.

Feeling that they are able to open up to educational professionals within schools and colleges about their status as a young carer. Realising that it isn’t about being ‘labelled’ but actually so they can receive the right support if they need it. Especially at college level, I think there is a lack of understanding about being a young carer and that they are able to access further support. (Manager, higher education organisation, survey ID98)

Young carers advocated for more time and space to deal with grief and other emotions when caring and during bereavement, including reduced hours, and a quiet room for independent study if needed, with a positive example given of the difference that this made to YC8 when she was eventually allowed this.

I know myself that some days where if I didn’t feel very well or if I was particularly anxious and I knew that I was going to have these awful lessons with this teacher that really didn’t get it, I just wouldn’t go. Whereas if I’d have known that actually I could have gone somewhere else and on the days where I wasn’t feeling great, that there were other places I could do the learning, then I probably would have attended more. Well, I did once I got to use the SDC (wellbeing/study room). (YCFG3, sister, YC8)

To support their transition post-education/into adult life, young carer participants also wanted a way of sharing information about their carer status, alongside greater understanding from employers and/or Job Centres, on how these experiences affected them. It was felt that whether in school, employment, or in the Job Centre, removing the need to regularly repeat their stories, relive their experiences and justify themselves, would reduce the emotional toll and effort required to engage. In addition to the life-skills training and guidance already noted, they also advocated for support with making future plans, recognising how lives are put on hold at this pivotal and transitional age between education and employment, childhood and adulthood. Check-in calls from somebody in the community were also suggested as a way of preventing those who have left education from ‘slipping through the net’, whilst also tackling problems with invisibility and social isolation for those living alone.

Since my mum died during my A levels, I didn’t get good A levels at all . . . So you know, job prospects at least right now don’t feel viable. So for a while I’ve been going to the Job Centre, claiming Universal Credit. But the problem with how the Job Centre works is that I get – I can get – a different job coach every single week. And it’s so tiring, every single week, having to have the conversation with the job coach . . . if it could just be almost like on like a file or something . . . I’m tired of justifying why my life has stopped for the past few years, every single time. (YCFG1, son, YC5)

## Discussion

This research illustrates the many challenges and needs of young people caring for terminally ill family members and during bereavement, from the perspectives of bereaved young people and the professionals supporting them. These challenges are reflected in three broad themes; providing care at the end of life, navigating bereavement and managing everyday life and relationships. Cutting across these themes are different, multi-faceted aspects of loss and change, and inadequacies in the support and guidance available to current and bereaved young/young adult carers, with important implications for theory, policy and practice.

### Relevance of the Dual Process Model of coping with bereavement

**Figure 1.**
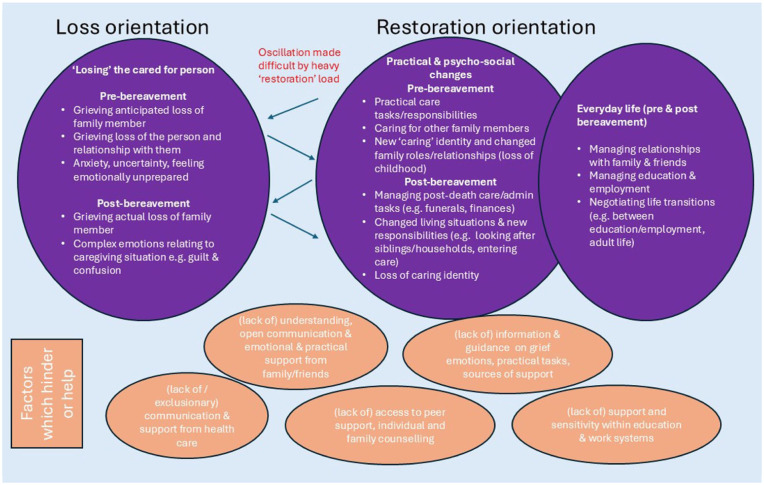
DPM and the caregiving and bereavement experiences of young/young adult carers.

Although the DPM was originally developed as a framework for conceptualising post-bereavement adult coping, the similar duality that we observed within many of our themes/sub-themes suggests the applicability of the model to young/young adult carers, pre- and post-bereavement.^41,42^ Here we discuss our themes, with other research, according to the model’s loss and restoration orientations, whilst also considering factors which hinder or help (Figure 1). In illustrating the strong relational aspects of these experiences, our adaptation of the model is more socially-situated than the original,^41,42^ extending its conceptual as well as contextual scope, and aligning with more critical perspectives which emphasise the social and relational contexts of caregiving, grief and bereavement,^40,49–51^ and the recognition given to ‘family-level’ coping in a later revision to the DPM.^52^

### Loss-oriented challenges and concerns

Young carers experience a number of loss-oriented challenges pre- and post-bereavement. In addition to grieving the loss of their cared-for relation, in response to their death or anticipated death,^45^ pre-bereavement grief is also felt in relation to the loss of the person and relationship with them, due to the changes brought about by their deteriorating health. This is well-evidenced in relation to dementia grief,^53^ but has also been described with regards to the loss of expected parenting roles/relationships amongst caregivers of children with life-limiting conditions,^44^ and in the disruption to existing and expected child-parent relations that has been documented in families with a terminally ill parent,^40^ with this research extending the evidence on this type of loss experience amongst young carers in end-of-life contexts.

Pre-bereavement young carers can feel emotionally unprepared and experience significant anxiety, exacerbated by the uncertainty and unpredictability of this phase of illness, and a lack of honest conversations within families and with healthcare professionals, as previously documented.^17,18,29,36–39^ Post-bereavement, complex emotions include feelings of guilt and confusion relating to whether more could have been done, relief that their relative’s suffering and their responsibilities have ended, and feeling happy again – providing new insights into these emotions and their similarities with those of bereaved adult caregivers.^3,4,50,51^ As in other research with bereaved young people,^29,30,33^ support from family and friends is important for managing and processing the emotions of grief, but also commonly lacking or unhelpful, reflecting a lack of understanding, differing grief responses within families and tendencies to hide feelings as a way of protecting other family members.

### Restoration-oriented challenges and concerns

Reflecting a restoration orientation,^41,42^ young carers also need to navigate the many practical and psychosocial changes and challenges that evolve across their caregiving and bereavement journeys. Pre-bereavement, these include significant responsibilities for their family members care and feeling unprepared for the practical tasks that accompany the end of life and death, with associated feelings of stress and anxiety compounded by lack of access to appropriate healthcare and/or inadequate or exclusionary communications from healthcare teams.^8,16,18,54,55^ Young carers commonly also take on caring responsibilities for siblings, as financial pressures necessitate that adult family members continue working.^17,29,44^ These more practical ‘lifestyle’ changes also have psychosocial consequences as the responsibilities of caring, and changes in family roles and relationships contribute to a changed identity and sense of self. Young carers commonly experience a sense of growing up too fast and missing out on their childhood due to these additional roles and responsibilities,^17,29^ while their ‘caregiver’ identities can be threatened by exclusionary interactions with healthcare staff and lack of recognition of their caregiving roles. This somewhat paradoxical situation has been described elsewhere in relation to mostly chronic conditions, whereby young carers can experience difficulties establishing a positive self-identity and fulfilling their caring duties due to contradictions with socially accepted constructions of childhood, which in turn influence the attitudes and behaviours of those around them and the extent to which they recognise and support young carers in their roles.^19^

In particular, this research provides new evidence on the practical challenges that are experienced post-bereavement, as young carers take on a different set of care tasks and responsibilities relating to after-death arrangements (e.g. funerals, administrative and financial matters), often with minimal guidance or support, and whilst grappling with the difficult emotions of grief. Living arrangements can change dramatically; young carers may need to go into care, while young adult carers may take on responsibilities for managing households and/or younger siblings, often feeling unprepared for this rapid transition to adult life. As described in a study of bereaved adolescent carers in Nigeria, they are also more likely to experience financial difficulties, uncertainty regarding their future and a sense of missed opportunities.^45^ The abrupt changes to daily life and routines brought about by the death are reflected in significant psychosocial disruption and loss of caring identities and roles, with this research again illustrating some of the similarities shared with bereaved adult caregiver experiences,^3,4,51^ but less documented for bereaved young carer populations.

As well as navigating the practical and psychosocial changes brought on by caring and then bereavement, young carers also need to manage other aspects of everyday life such as relationships, social life and education, also situated within the ‘restoration orientation’ of the DPM.^41,42^ While the benefits of mutual support and understanding within families and peer-groups are evident, so too are difficult family coping and grief dynamics, and the lack of support and understanding within friend-networks, illustrating the strong relational contexts shaping experiences of caregiving and grieving.^40,49–52^ These difficulties not only limit opportunities for sharing and processing feelings, but also leave young people feeling lonely and isolated.^29,30^ As argued by Turner and Almack,^40^ young carers may also need to negotiate the ‘moral breach’ and associated psychosocial disruption created by families prioritising the care needs of the sick parent over those of their children.

Education experiences were also widely recognised to be impacted by the practical and emotional challenges of caring and bereavement.^23,34^ Attending and completing school or college work is made difficult by the practical demands of caring for family members, grief and the complex emotions which continue following the death. These difficulties are exacerbated by unsupportive school/college environments, which include problems with bullying and lack of understanding, sensitivity or flexibility amongst school/college staff or in school policies.^30,34^ Inconsistency in the specialist support available within schools was noted,^30^ while the reluctance of young carers/families to disclose their caring status due to stigma, a desire to protect family members and lack of self-awareness of their caring situations/entitlements further limits access to available support.^17^ The added challenges of negotiating life-stage transitions, in the midst of caring and grief were also highlighted, providing illustrative examples of this previously observed phenomenon.^17^ In this study, transitioning between education and (un)employment was suggested to be particularly challenging for young adult carers, who find it difficult to plan for the future when in emotional turmoil or lacking adult guidance, find work in the context of poor educational outcomes, and miss out on institutional support if not in education or stable employment.

### Difficult oscillation

With regards to the construct of ‘oscillation’,^41,42^ the heavy ‘restoration’ demands of pre- and post-death care tasks and managing education, alongside unhelpful pressure from others to engage in ‘healing’ activities, could be seen to undermine oscillation between the two orientations. Young carers reflected on how these pressures impeded their abilities to focus on and have control over their grief and loss-related emotions, sharing similarities with the difficult oscillation described for Nigerian HIV bereaved adolescents who experienced delayed grief due to the demands of caregiving,^45^ with such experiences potentially suggestive of a mechanism for disenfranchised grief for these groups of young people.^56^

### Strengths, limitations and implications for further research

This research engaged with a wide variety of professionals working with young/young adult carers, while the inclusion of nine young people with recent caregiving and bereavement experiences contributed vital, less heard perspectives and experiences. Although two of the young carer groups were very small, this enabled in-depth exploration of individual experiences and perspectives, which complemented and provided more nuanced examples to the broader perspectives of professionals which drew on their experiences of supporting large numbers of young people. The reflective contributions of team members with closely relevant personal and professional experiences to data-generation and analysis also significantly strengthened these processes and their outputs, including helping to address possible limitations caused to the survey by the absence of pilot-testing.

However, despite extensive recruitment efforts we did not recruit as many young people as we had planned, and no participants were aged under 18. The heterogeneity in these groups in terms of relationship to the person who died, their illness/condition and related caregiving trajectories also meant that it was not possible to explore group differences according to these salient characteristics. Future research should focus on engaging with younger age groups, and where possible aim for more homogenous group compositions, or larger group-sizes across the study sample. It is also likely that the online and group-based methods used in this study may not have appealed to some young people; providing a choice of interviews or focus groups, in-person or online formats, are recommended to help increase recruitment.

### Implications for policy and practice

In illustrating the social/relational, community and healthcare contexts shaping the caregiving and bereavement experiences of young/young adult carers, this research closely aligns with public health approaches to dying, death and bereavement,^57^ and points to various cross-sector changes needed to improve the informal and formal sources of support which help them to cope.

The described limitations within education settings points strongly to the case for school/college-based improvements. While there have been positive changes recently introduced to improve school- and college-based support for young carers in England, such as inclusion in the Schools Census^58^ and Ofsted inspection framework,^59^ further changes are needed. This includes staff training which fosters greater awareness and sensitivity amongst staff to the circumstances and needs of current and bereaved young carers (including how to identify them), alongside greater allowances and flexibility in school policies with regards to attendance and managing school-work and assessments. Improved communication and information-sharing within and across institutions is needed to limit the ‘retelling’ of stories and enable the greater understanding needed from transitional organisations such as job centres, employers, universities/further education colleges. Greater access to specialist support within schools/colleges in underserved localities is also necessary to address the observed inconsistencies and inequities. This is consistent with the recommendations of the UK Commission on Bereavement (UKCB) for the implementation of a bereavement policy, staff training, and a process for supporting a bereaved child or young person and their family in all educational settings,^30^ whilst also pointing to the potential benefits of holistic policies and plans which cover pre- and post-bereavement.

Improving death and grief literacy at population level offers another means of improving the informal support available to pre- and post-bereaved young people and their families, as well as personal preparedness for coping with death and bereavement. Recently introduced in the Relationships, Sex and Health Education (RSHE) curriculum for England,^60^ school-based grief education can facilitate more compassionate school environments, as well as societies longer term,^30,34,61^ increasing community capacity to support young (and older) populations facing dying, death and bereavement. Death and grief literacy/education interventions should also be delivered more widely through workshops and events, and public information campaigns – about and for young people – extending the focus of public health palliative care and compassionate communities approaches.^30,62^

Young carer projects and initiatives providing opportunities for social activities and connection within local communities are also essential for tackling social isolation, strengthening social support and providing breaks from caring, and alongside schools/colleges, may also be well placed to provide the requested information and training on general life skills and planning for the future. Introducing designated points of contact within communities would be especially helpful for recently bereaved young adult carers, particularly those living alone and/or responsible for siblings, and at risk of ‘slipping through the net’ if they are no longer in education or stable employment. Likewise, a commitment from/support for employers to develop more compassionate workplaces, including clear policies and well-publicised access to adequate carer and bereavement leave for all employees, would also benefit those who are working.^30^

Across health and social care, increased awareness and identification of young carers is needed, including greater implementation of statutory young carer assessments,^7^ and incorporation in the standard assessment practices of healthcare professionals supporting families at the end of life.^1,5^ In healthcare, formal assessments should be accompanied by improved communication between healthcare professionals and young carers, and support that facilitates open and honest discussions within families as the end-of-life approaches, not only as a way of improving preparedness, but also the mutual support available within families.^20,38,39,63^ There also needs to be better information and guidance given to young carers and their families on the more practical aspects of preparing for, and managing a bereavement (e.g. wills, funerals and financial affairs), as well as the emotions of grief, and opportunities for (and effective signposting to) peer support and individual and family counselling pre- and post-bereavement.^17,20,29,30^

## Conclusion

Young/young adult carers experience multiple challenges when providing care at the end of life, when navigating their bereavement and more generally managing relationships and everyday life. Experiences of loss and change are multi-faceted, and exacerbated by various limitations in the guidance and support available pre- and post-bereavement. This research suggests the theoretical utility of a more socially situated application of the DPM for conceptualising the pre- and post-bereavement experiences of young/young adult carers. Cross-sector changes, reflecting public health approaches, are needed to improve the informal and formal support available to young people when caring and bereaved.

## Supplemental Material

sj-docx-3-pcr-10.1177_26323524261442209 – Supplemental material for ‘I’m tired of justifying why my life has stopped’: A qualitative analysis of the experiences and support needs of young people caring at the end of life and into bereavement Supplemental material, sj-docx-3-pcr-10.1177_26323524261442209 for ‘I’m tired of justifying why my life has stopped’: A qualitative analysis of the experiences and support needs of young people caring at the end of life and into bereavement by Emily Harrop, Zoe Abbott, Noreen Hopewell-Kelly, Silvia Goss, Charlie Byrne, Jane Bayliss, Kerry Jones, Oliver Clabburn, Rachel Warren, Alison Penny and Helen Leadbitter in Palliative Care and Social Practice

sj-pdf-1-pcr-10.1177_26323524261442209 – Supplemental material for ‘I’m tired of justifying why my life has stopped’: A qualitative analysis of the experiences and support needs of young people caring at the end of life and into bereavement Supplemental material, sj-pdf-1-pcr-10.1177_26323524261442209 for ‘I’m tired of justifying why my life has stopped’: A qualitative analysis of the experiences and support needs of young people caring at the end of life and into bereavement by Emily Harrop, Zoe Abbott, Noreen Hopewell-Kelly, Silvia Goss, Charlie Byrne, Jane Bayliss, Kerry Jones, Oliver Clabburn, Rachel Warren, Alison Penny and Helen Leadbitter in Palliative Care and Social Practice

sj-pdf-2-pcr-10.1177_26323524261442209 – Supplemental material for ‘I’m tired of justifying why my life has stopped’: A qualitative analysis of the experiences and support needs of young people caring at the end of life and into bereavement Supplemental material, sj-pdf-2-pcr-10.1177_26323524261442209 for ‘I’m tired of justifying why my life has stopped’: A qualitative analysis of the experiences and support needs of young people caring at the end of life and into bereavement by Emily Harrop, Zoe Abbott, Noreen Hopewell-Kelly, Silvia Goss, Charlie Byrne, Jane Bayliss, Kerry Jones, Oliver Clabburn, Rachel Warren, Alison Penny and Helen Leadbitter in Palliative Care and Social Practice
