# Supplementary material for: ‘I’m tired of justifying why my life has stopped’: A qualitative analysis of the experiences and support needs of young people caring at the end of life and into bereavement
Source: Palliat Care Soc Pract. 2026 May 12;20:26323524261442209. doi: 10.1177/26323524261442209 (PMC13172680; doi:10.1177/26323524261442209)
Supplement: sj-docx-3-pcr-10.1177_26323524261442209 – Supplemental material for ‘I’m tired of justifying why my life has stopped’: A qualitative analysis of the experiences and support needs of young people caring at the end of life and into bereavement [file sj-docx-3-pcr-10.1177_26323524261442209.docx]

**Supporting young carers and young adult carers at the end of life and into bereavement: a mixed-methods study**

**Focus group guide-Young Carer groups**

**Introduction**

The focus group discussion is going to explore your views and experiences of the support given to young people who are looking after someone at the end of their life and into bereavement. We are going to be exploring some of the challenges young people face in getting support but also talk about what good support looked/looks like. By the end of the group, we will also try to identify what support is most needed and how the needs of young people might be better met by services.

We know that some of this may be difficult to talk about and if you feel like you want to take a break or stop altogether that is absolutely fine- if you do leave the call one of us will follow up with you to make sure that you are ok. Or if there is anything you don’t want to talk about that is fine too, and you are welcome to use the chat as well if there are some things that you would find easier to type than say.

Reminder about confidentiality and anonymity when reporting

**Start recording with consent**

**Introductions**- name, age, who you cared for and why you wanted to take part

We will start off thinking about the challenges that young people face and the support that they need when caring and then will go onto talk about support needs during bereavement

**Part 1: Caring for a person at end of life.**

**Read vignette 1: caring at the end of life (Hannah’s story)**

1. Are there particular features of Hannah’s story that struck you? How does it compare to your own experiences?
2. What do you think are the main challenges that young people like yourselves and Hannah can face when they are looking after someone who is approaching the end of their life? (prompts; physical care, emotional, managing other areas of life e.g. education, social life)
3. What different types of support were you offered when you were in this situation? (prompt-health care, education, young carers organisations, friends & family)
4. In what way was the support helpful or not helpful?
5. What other types of support did you or other young people in similar situations that you may know have needed?
6. How easy/hard was it get the support that you needed?

**Part 2: Bereavement**

**Read Vignette 2:** Post caring loss, grief and bereavement (Dan’s story)

1. Are there particular features of Dan’s story that struck you? How does it compare to your own experiences?
2. What do you think are the main challenges that young people like yourselves and Dan can experience when the person they were caring for dies? (prompts- emotional, practical, financial, relationships with friends & family)
3. What support did you receive when you experienced the bereavement? ? (prompts; healthcare, education, young carer or bereavement organisations)
4. How helpful did you find this support?
5. Were there things that would have been helpful at that time that you weren’t offered?

**Support services**

1. What do the professionals/volunteers who are providing support to young people and their families need to know about being a young carer when they are caring or in bereavement?
2. What additional support/resources/ help would you put in place for young people looking after someone at the end of their life and when they are bereaved?
3. Is there anything else that you would like to share that you think is important for us to know.

**Close:**

- Thank you for your time and help and for sharing your thoughts and experiences.
- Would you like to receive a summary of results when available
- How did you find taking part today? If there is anything you would like to talk about privately please let us know and we can either stay on or arrange another call. Our contact details are on the information sheet if you would like to get in contact for any reason – there are also details of bereavement support
- What nice things do you have planned for the remainder of the evening/weekend?
- We will be in touch by e-mail with your voucher codes.

**Focus group guide for professionals working with young carer groups**

**Introduction**

The focus group discussion is going to explore your views and experiences of the support given to young people who are looking after someone who is terminally ill and into bereavement. We are going to be exploring some of the challenges young people face in getting support but also talk about what good support looked/looks like. By the end of the group, we will also try to identify what support is most needed and how the needs of young people might be better met by services.

Reminder about confidentiality and anonymity when reporting

**Start recording with consent**

**Introductions**- name, what their organisation does, what they do?

**Support needs and provision**

1. What are the main ways in which you support young carers who are looking after someone who is terminally ill and/or when they are bereaved ?
2. What do you see to be the main difficulties and needs for support that are experienced by the young people that you work with a) when they are caring for a terminally ill family member and b) when they are bereaved? (prompts; emotional, practical, education/employment- check that EoL and bereavement covered)
3. What do you find to be the main challenges that you face when supporting these groups of young people and trying to meet these needs?
4. Do you have any examples of specific ways in which you’ve responded to or tried to address these difficulties? E.g. Resources or initiatives that you or your organisation have put in place to help support young carers who are looking after someone who is terminally ill and/or bereaved.
5. What additional support/resources/ help are needed to improve the support available for young people looking after someone who is terminally ill and when they are bereaved?

- resources, policy, legislation

1. What would help you and/or your organisation improve the support you provide?

What would any of this look like

**Accessing support**

1. What do you think are the main challenges that young people face in accessing the types of support we’ve talked about when they are caring or are bereaved (prompts: awareness, signposting/information, identification, stigma).
2. Which groups of young carers are we missing? How might we improve access to support generally and specifically with these groups?
3. Is there anything else that anyone would like to add?.

**Close/ thank you!**
