# Supplementary material for: ‘I’m tired of justifying why my life has stopped’: A qualitative analysis of the experiences and support needs of young people caring at the end of life and into bereavement
Source: Palliat Care Soc Pract. 2026 May 12;20:26323524261442209. doi: 10.1177/26323524261442209 (PMC13172680; doi:10.1177/26323524261442209)
Supplement: sj-pdf-2-pcr-10.1177_26323524261442209 – Supplemental material for ‘I’m tired of justifying why my life has stopped’: A qualitative analysis of the experiences and support needs of young people caring at the end of life and into bereavement [file sj-pdf-2-pcr-10.1177_26323524261442209.pdf]

Supporting young carers and young adult carers through caring at the end of life and into bereavement

Study information and consent to participate

Thank you for considering taking part in this survey. We appreciate that this is an especially busy and pressured time for all those working in the public and voluntary sector and are very grateful for your help.

WHO SHOULD COMPLETE THIS SURVEY?

This survey is for anyone in a voluntary or professional role who has experience of supporting or working with children and young people who are young carers (under 18) or young adult carers (18-25) and their families, when they are caring or in bereavement. We are keen to hear from people working or volunteering with relevant experience in different roles in health and social care, education and the voluntary sectors.

The information collected in this survey will be used to inform policy, practice and future research.

ABOUT THIS SURVEY

Background: Family members often play a vital role in providing care for relatives living with long-term conditions and terminal illnesses. In some families, much of this care is provided by young carers or young adult carers who may provide care for e.g. a parent, sibling or grandparent. We know that young carers and young adult carers can be significantly impacted by their caring roles and responsibilities and that they have different experiences and needs to older adult carers, including during bereavement. More research is needed to better understand how to improve the support provided to young people during these difficult times in their lives.

This project explores experiences, support needs and support provision for young/young adult carers who are caring for a relative who is terminally ill, and when they become bereaved.

The study is funded by Marie Curie and led by researchers at Cardiff University, in collaboration with the Young Carers Initiative and the Open University.

Purpose of the survey: This scoping survey aims to understand the wide range of cross-sector support provided to current and bereaved young carers and young adult carers in the UK, including gaps and challenges in service provision, useful initiatives and resources and priorities for change. The results will help us to identify ways to improve the support that is currently available to young/young adult carers, and highlight areas for future research. They will also enable us to share learning and knowledge across different professional groups and organisations, including policy and practice recommendations for health, social care and voluntary sector organisations and professionals.

DATA PROTECTION

Before you decide whether to consent to participate, please read this page.

Voluntary participation: Participation is voluntary, and you may choose not to answer some, or any, of the survey questions. If you decide to withdraw at any stage, you do not have to give a reason why. Simply close your web browser to exit the survey.

Confidentiality: The information you provide will only be seen by the project staff who are undertaking this study. Any information we publish that might identify you, or others, will be anonymised.

Data protection and usage: All data collected in this survey will be held securely at Cardiff University in accordance with the General Data Protection Regulations (GDPR; EU 2016/679). For more information on data protection, please follow the link: General Data Protection Regulation (GDPR; EU 2016/679). Cookies, personal data stored by your web browser, are not used in this survey.

By participating in this survey, you are agreeing that your survey responses and anonymised extracts of text may be included in future reports, professional journals and future research. The results will be disseminated via e.g. newsletters, blogs, webinars, workshops, conferences, peer-reviewed journal articles, press releases and social media. The anonymised data may be archived in a data-repository and might be used in secondary analysis or for educational purposes in the future. Your personal data (identifying information) will be kept securely for 5 years after the research is completed in line with GDPR and Cardiff University policies. The anonymised data-sets and study results are kept at Cardiff University for at least 5 years after they are published: https://www.cardiff.ac.uk/public-information/policies-and-procedures/data-protection/research-participants-data-protection-notice.

You can ask for all your personal details to be erased as well as your responses. However, this right may be restricted in order to ensure the integrity of the research in accordance with the General Data Protection Regulations (GDPR) and Data Protection Act 2018 (https://www.gov.uk/data-protection).

We need to manage your records in specific ways for the research to be reliable, which means that we won't be able to let you change the data we hold about you or withdraw data that has already been anonymised and analysed. If you would like to withdraw your data after completing this survey, please contact the researcher using the details below.

Risks and benefits: When using the internet, there can be a risk of compromising privacy, confidentiality and/or anonymity. We are using a secure platform to conduct the survey to minimise these risks. By completing this survey, you are helping understand the support currently available to young carers/young adult carers and what changes and improvements are needed.

Contact information: If you have any concerns or questions relating to this survey, please contact Dr Silvia Goss, Research Assistant at gosss1@cardiff.ac.uk.

Additionally, if you have a concern about the conduct of the study, please contact Dr Emily Harrop at harrope@cardiff.ac.uk, Marie Curie Research Centre, Cardiff University, Neuadd Meirionnydd, Heath Park, CF14 4YS. If you have a complaint about the study, please contact the University's Research Ethics team by emailing medic_rec@cardiff.ac.uk. Complaints or concerns are taken seriously and will be addressed immediately.

INSTRUCTIONS FOR COMPLETION

- The survey should take 10-20 minutes to complete
- Please add any comments in the blank boxes that you feel are relevant or important.
- The survey is designed to capture information on many different types of services and some questions may appear less relevant to you. We would be grateful if you could please try to answer all questions that apply to you.
- You can save your responses and return to complete your survey later. To do so, please click on the 'finish later' link at the bottom of the page. You will then need to either bookmark your unique URL link in your browser or ask for it to be emailed to you.
- You can navigate the survey and edit your answers up to the point you click the 'finish survey' button. To go back in the survey (e.g. to revise a response), please click the 'previous' button at the bottom of the page.

Consent

By agreeing to participate in this survey, you indicate that you have read and understood the information provided on the previous page and that you are aged 16 or over.

I consent to participate in this survey, and I agree that I have read and understood the information provided provided above. *

- ◯ Yes
- ◯ No

Background information about your role and the service/organisation you work or volunteer for

Your job title:

Name of your service/organisation:

If your service is a local branch of a national organisation/charity (e.g. Cruse, MIND, Place2Be), please state the name of that organisation:

Background information about your role and the service/organisation you work or volunteer for

What kind of catchment area do you serve?

- ◯ National
- ◯ Regional
- ◯ County
- ◯ Other specific catchment area

Please specify:

- ☐ UK
- ☐ England
- ☐ Wales
- ☐ Scotland
- ☐ Northern Ireland

Please specify:

- ☐ North East England
- ☐ North West England
- ☐ Yorkshire & the Humber
- ☐ East Midlands
- ☐ West Midlands
- ☐ East of England
- ☐ Greater London
- ☐ South East England
- ☐ South West England
- ☐ Wales
- ☐ Scotland
- ☐ Northern Ireland

Please specify if you selected 'County':

Please specify if you selected 'Other specific catchment area':

Background information about your role and the service/organisation you work or volunteer for

Which of the following best describes your organisation?

- ◯ National bereavement charity / Non-governmental Organisation (NGO)
- ◯ Branch of national bereavement charity / NGO (e.g. SOBS area, Cruse branch)
- ◯ Local bereavement charity / NGO
- ◯ National Carers/Young Carers charity / NGO
- ◯ Branch of national Carers/Young Carers charity / NGO
- ◯ Local Carers/Young Carers charity / NGO
- ◯ Other national charity (e.g. MIND, Age UK, Place2Be, Marie Curie)
- ◯ Branch of other national charity / NGO (e.g. MIND, Age UK, Place2Be, Marie Curie)
- ◯ Other local charity / NGO
- ◯ Hospice/community palliative care team
- ◯ Hospice bereavement service
- ◯ NHS Children or Adults Mental Health Services (e.g. CAMHS)
- ◯ Other NHS specialist service
- ◯ NHS primary care team/GP practice
- ◯ School/College or other educational institution
- ◯ Local authority social work team
- ◯ Funeral care service
- ◯ Faith-based organisation
- ◯ Other

Please specify if you selected 'Other NHS specialist service' above:

Please specify if you selected 'Other' above:

Background information about the service/organisation you work or volunteer for

What age groups are the primary focus of your support? Tick all that apply:

- [ ] Children and young people (aged under 18)
- [ ] Young adults aged 18-25
- [ ] Adults
- [ ] All
- [ ] Other

Please specify if you selected 'Other':

Does your organisation, project or role have a specific or specialist focus on young carers or young adult carers? (tick all that apply)

- [ ] Current young carers (aged under 18)
- [ ] Bereaved young carers (aged under 18)
- [ ] Current young adult carers (aged 18+)
- [ ] Bereaved young adult carers (aged 18+)
- [ ] Not a specific focus of my role or organisation

Since you indicated that your organisation, project or role focuses on young carers under 18: What age do you provide support from? (i.e the youngest age you support)

Since you indicated that your organisation, project or role focuses on young adult carers aged 18 and older: What age do you provide support until? (i.e. the oldest age you support)

Background information about the service/organisation you work or volunteer for

Does your organisation, project or role have a specific or specialist focus on particular illnesses or conditions?

- ◯ Yes-Cancer
- ◯ Yes-Non-cancer
- ◯ No

Please specify which particular type(s) of cancer:

Please specify which non-cancer illness(es) or condition(s):

Approximately how many young carers/young adult carers does your organisation typically support each year?

- ◯ Up to 20
- ◯ 21-40
- ◯ 41-60
- ◯ 61-80
- ◯ 81 or over
- ◯ I don’t know

Information about the support you and/or your organisation provide and how

Please indicate which of the below types of support you and/or your organisation provide and how it is provided. Please tick all that apply:

Support with educational needs and pastoral care e.g. at school

- [ ] Resources (e.g. written, visual, audio)
- [ ] In-person support
- [ ] Remote/virtual support
- [ ] Not a type of support or information we provide
- [ ] Don't know

Support with practical aspects of caregiving e.g. administering medication, diet, other physical care tasks

- [ ] Resources (e.g. written, visual, audio)
- [ ] In-person support
- [ ] Remote/virtual support
- [ ] Not a type of support or information we provide
- [ ] Don't know

Emotional aspects of preparing for death and bereavement e.g. anticipatory grief

- [ ] Resources (e.g. written, visual, audio)
- [ ] In-person support
- [ ] Remote/virtual support
- [ ] Not a type of support or information we provide
- [ ] Don't know

Practical aspects of preparing for death and bereavement e.g. advance care plans, funerals, financial support

- [ ] Resources (e.g. written, visual, audio)
- [ ] In-person support
- [ ] Remote/virtual support
- [ ] Not a type of support or information we provide
- [ ] Don't know

Does your organisation offer respite opportunities and breaks from caregiving for young carers and adult young carers?

- ○ Yes

- ○ No
- ○ Does not apply
- ○ Don't know

Please indicate which of the below types of group-based or one-to-one/family support you and/or your organisation provide pre- and/or post-bereavement and how it is provided. Please tick all that apply.

Informal peer support with other children and young people in caregiving roles/who have close family members

who are terminally ill

- [ ] In-person support
- [ ] Remote/virtual support
- [ ] Not a type of support or information we provide
- [ ] Don't know

Informal peer support with other bereaved children and young people

- [ ] In-person support
- [ ] Remote/virtual support
- [ ] Not a type of support or information we provide
- [ ] Don't know

Group-based support facilitated by someone with training (e.g. group counselling) - pre-bereavement

- [ ] In-person support
- [ ] Remote/virtual support
- [ ] Not a type of support or information we provide
- [ ] Don't know

Group-based support facilitated by someone with training (e.g. group counselling) - post-bereavement

- [ ] In-person support
- [ ] Remote/virtual support
- [ ] Not a type of support or information we provide
- [ ] Don't know

One-to-one or family support (e.g. individual or family counselling by someone with training) - pre-bereavement

- [ ] In-person support
- [ ] Remote/virtual support
- [ ] Not a type of support or information we provide
- [ ] Don't know

One-to-one or family support (e.g. individual or family counselling by someone with training) - post-bereavement

- [ ] In-person support
- [ ] Remote/virtual support
- [ ] Not a type of support or information we provide
- [ ] Don't know

Specialist mental health or psychological support - pre-bereavement

- [ ] In-person support
- [ ] Remote/virtual support

- [ ] Not a type of support or information we provide
- [ ] Don't know

Specialist mental health or psychological support - post-bereavement

- [ ] In-person support
- [ ] Remote/virtual support
- [ ] Not a type of support or information we provide
- [ ] Don't know

Please provide further details on the main ways in which you support young carers/young adult carers during caregiving and/or in bereavement.

Your views on unmet support needs and challenges faced by young carers/adult young carers

What do you see as the main challenges or unmet needs for support young carers/young adult carers face when caring for someone at the end of life and/or when they are bereaved following the person's death?

Do you think there are specific groups of young carers/young adult carers with unmet support needs that may experience particular challenges or barriers to accessing support?

- ○ Yes
- ○ No
- ○ Don't know

If you selected 'yes' above, please indicate here which specific groups of young carers/young adult carers may experience particular challenges or barriers, when caring or in bereavement. Please select all that apply.

- ☐ Young/ young adult carers from lower socioeconomic groups/poorer areas
- ☐ Young carers/adult carers from ethnic minority or particular faith communities
- ☐ Male young/young adult carers
- ☐ Female young/young adult carers
- ☐ Young children (12 and under)
- ☐ Adolescents (13-17)
- ☐ Young adults (18+)
- ☐ Young carers/young adult carers who are neurodiverse or have additional learning needs
- ☐ Young carers/young adult carers with mental health conditions
- ☐ Young carers/young adult carers in refugee/migrant/asylum-seeking families
- ☐ Young/ young adult carers in single-parent families
- ☐ Young carers/young adult carers in Armed Forces families
- ☐ Young/ young adult carers for people with specific conditions

☐ Other

If you selected 'Young carers/adult carers from ethnic minority or particular faith communities', please specify here:

If you selected 'Young/ young adult carers for people with specific conditions' above, please state which condition(s):

If you selected 'Other', please specify here:

Based on your experience, please tell us more about the specific needs/challenges faced by any of these groups.

Your views on the challenges organisations/services face when supporting young carers/adult young carers

What challenges do you/your organisation currently face in relation to service delivery and your work with young/young adult carers? Please tick all that apply.

- ☐ Access to funding
- ☐ Charitable objectives restricting scope of support offered (e.g. only able to work with cancer caregivers)
- ☐ Funding conditions limiting the duration of support offered (e.g. only pre-bereavement support)
- ☐ Access to training
- ☐ Lack of guidance or specific tools/resources to inform work with young/young adult carers (e.g. needs assessment)
- ☐ Difficulty understanding or implementing relevant legislation (e.g. Children & Families Act 2014, Care Act 2014)
- ☐ Access to specialist support to refer on to e.g. mental health services
- ☐ Lack of capacity: not enough volunteers/staff available to work
- ☐ Unable to meet demand for support/service
- ☐ Unable to meet the emotional support needs of young/young adult carers
- ☐ Unable to meet the practical support needs of young/young adult carers
- ☐ Difficulty providing continuity of care pre- and post-bereavement
- ☐ Difficulty maintaining support through other life transitions e.g. finishing school, University, reaching ‘adulthood’
- ☐ Unable to provide transport to young carers/adult young carers to access services/support
- ☐ Challenges related to safeguarding young carers/young adult carers
- ☐ Lack of guidance or specific tools/resources related to service impact and evaluation measurement
- ☐ Lack of awareness of available support (among e.g. young carers/adult young carers and their families, other services/organisations)
- ☐ Other

If you selected 'Other', please specify:

Please describe the most significant of these challenges that you/your organisation experience in providing support and what the reasons for this may be.

Your views on ways to improve the support available to young carers/adult young carers

Please tell us about any initiatives or resources that have helped you/your organisation with your work with young/young adult carers.

What initiatives, resources or changes in policy/legislation would you most like to see to help you improve the support that you are able to give to young carers/adult carers during the end of life of their relative and/or in their bereavement?

What next?

Alongside our focus groups with bereaved young carers/young adult carers, we will also be organising two online focus groups with volunteers and/or professionals who completed this survey to explore their experiences and ideas for change in more depth.

The focus groups are expected to take place within the next two months. They will be run via Microsoft TEAMS and will last for approximately 60 minutes.

Would you be happy to be contacted by a researcher with further information about participating in one of our focus groups?

- ◯ Yes
- ◯ No

Thank you for your interest! Please provide us with your contact details below so we can contact with further information about taking part in one of our focus groups. Your name and email address will not be shared beyond the study team. If you have any further questions, please contact gosss1@cardiff.ac.uk.

Your name:

Your email address:

Your phone number:

What next?

We would also like to develop a network of people interested in future work and collaboration in this area.

Would you like to receive further updates and news from us including a summary of study results?

- ◯ Yes (summary of study results only)
- ◯ Yes (further updates, news and summary of results)
- ◯ No, thank you.

Thank you for your interest! Please provide us with your contact details below (if you have not already done so on the previous page) so we can contact you with the requested information in the future. Your name and email address will not be shared beyond the study team. If you have any further questions, please contact gosss1@cardiff.ac.uk.

Your name:

Your email address:

Your phone number:
